# Rapid Detection and Inhibition of SARS‐CoV‐2‐Spike Mutation‐Mediated Microthrombosis

**DOI:** 10.1002/advs.202103266

**Published:** 2021-10-23

**Authors:** Sandro Satta, Angela Lai, Susana Cavallero, Cayden Williamson, Justin Chen, Ana M. Blázquez‐Medela, Mehrdad Roustaei, Barbara J. Dillon, Nureddin Ashammakhi, Dino Di Carlo, Zhaoping Li, Ren Sun, Tzung K. Hsiai

**Affiliations:** ^1^ Division of Cardiology Department of Medicine David Geffen School of Medicine at University of California Los Angeles CA 90095 USA; ^2^ Department of Medicine Veterans Affairs Greater Los Angeles Healthcare System Los Angeles CA 90073 USA; ^3^ Department of Bioengineering Henry Samueli School of Engineering & Applied Science University of California Los Angeles CA 90095 USA; ^4^ Division of Clinical Nutrition Department of Medicine David Geffen School of Medicine at University of California Los Angeles CA 90095 USA; ^5^ Department of Molecular and Medical Pharmacology David Geffen School of Medicine at University of California Los Angeles CA 90095 USA; ^6^ School of Biomedical Sciences Li Ka Shing Faculty of Medicine The University of Hong Kong Hong Kong China

**Keywords:** inflammation, microfluidic chip, SARS‐CoV‐2, thrombosis

## Abstract

Activation of endothelial cells following severe acute respiratory syndrome coronavirus 2 (SARS‐CoV‐2) infection is thought to be the primary driver for the increasingly recognized thrombotic complications in coronavirus disease 2019 patients, potentially due to the SARS‐CoV‐2 Spike protein binding to the human angiotensin‐converting enzyme 2 (hACE2). Vaccination therapies use the same Spike sequence or protein to boost host immune response as a protective mechanism against SARS‐CoV‐2 infection. As a result, cases of thrombotic events are reported following vaccination. Although vaccines are generally considered safe, due to genetic heterogeneity, age, or the presence of comorbidities in the population worldwide, the prediction of severe adverse outcome in patients remains a challenge. To elucidate Spike proteins underlying patient‐specific‐vascular thrombosis, the human microcirculation environment is recapitulated using a novel microfluidic platform coated with human endothelial cells and exposed to patient specific whole blood. Here, the blood coagulation effect is tested after exposure to Spike protein in nanoparticles and Spike variant D614G in viral vectors and the results are corroborated using live SARS‐CoV‐2. Of note, two potential strategies are also examined to reduce blood clot formation, by using nanoliposome‐hACE2 and anti‐Interleukin (IL) 6 antibodies.

## Graphical abstract

SARS‐CoV‐2 infection drives the increasingly recognized thrombotic complications in COVID‐19 patients, potentially due to the SARS‐CoV‐2 Spike protein binding to human angiotensin‐converting enzyme 2. Vaccination therapies that are spike‐based can lead to a similar outcomes. Endothelialized channels exposed to patient specific blood in microfluidic platforms can be used to rapidly test for blood coagulation, predicting thrombotic events.

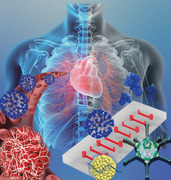

## 1 Introduction

Coronavirus disease 2019 (COVID‐19) has lingered with multiple resurgences and new variants, infecting more than 160 million people worldwide with severe acute respiratory syndrome coronavirus 2 (SARS‐CoV‐2).^[^ ^1^ ^]^ The current pandemic has stimulated the use of novel vaccines therapies, which have been developed with unprecedent speed; however, the rising SARS‐CoV‐2 mutations and wide spread of variants have delayed the full reoperation of the society. Thus, recapitulating patient‐specific whole blood with nanoliposomes to mimic viral variants or vaccinations is timely and clinically significant to predict and protect the vulnerable populations at risk of COVID‐19‐associated coagulation.

Despite the absence of major adverse events from the early clinical trials, complications developed from SARS‐CoV‐2‐based vaccinations are emerging worldwide,^[^ ^2^ ^]^ potentially in association with demographics and pre‐existing conditions.^[^ ^3^, ^4^ ^]^ Severe inflammatory reactions (e.g., myocarditis and pericarditis) and thrombosis with thrombocytopenia have been observed in vaccinated patients following viral vector vaccines (Vaxzevria, JNJ‐78436735) or the messenger RNA (mRNA)‐based vaccines (e.g., mRNA‐1273, BNT162b2). Furthermore, other type of vaccine technologies, such as synthetic nanoparticles (e.g., Novavax, EpiVacCorona), or DNA fragment (e.g., Inovio) are currently being tested in clinical trials.

Among the SARS‐CoV‐2 membrane proteins (M, N, E), Spike (S) protrudes from the virus and comprises two subunits: the S1 contains the receptor‐binding domain (RBD) and S2 subunit mediates the membrane fusion process.^[^ ^5^ ^]^ The S1 allows SARS‐CoV‐2 to bind the human angiotensin‐converting enzyme 2 receptors (hACE2) for further cellular internalization. For this reason, the Spike protein has received particular attention from both the scientific community and pharmaceutical industry in the development of the novel vaccines technologies.

**Figure 1:**
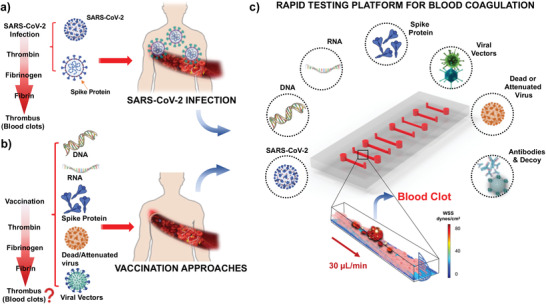
Rapid testing microfluidic platform for early thrombosis recapitulates SARS‐CoV‐2 and Spike‐mediated technologies thrombotic effect. a) Schematic of SARS‐CoV‐2 infection‐mediated thrombus formation in the microcirculation. In response to viral infection, inflammatory cells are recruited to activate the extrinsic and intrinsic coagulation pathways, leading to thrombin production. Thrombin cleaves fibrinogen to fibrin, which in turn, promotes platelet aggregation and fibrin deposition to form blood clots in the microcirculation. b) Similarly, SARS‐CoV‐2 vaccination therapies Spike‐based technologies have the potential role to increment blood coagulation. c) SARS‐CoV‐2, Spike protein, and Spike variant for mimicking microcirculation environment were assessed for their thrombotic phenotypes in multiple endothelialized microfluidic channels (2 cm x 400 mm x 100 mm). Antibody anti‐IL6 and decoy nanoliposome‐hACE2 were also tested together with the aforementioned conditions. SARS‐CoV‐2, Spike protein, and Spike variant expressed using viral vectors were incubated in the PDMS‐based microfluidic channels for 12 h at 37 °C, followed by a thrombosis assay in the presence of human subject‐specific whole blood at wall shear stress of 25 dyne cm^−2^. Thrombus formation was quantified in terms of fibrin and platelet deposition.

Following SARS‐CoV‐2 internalization to the host cells, S protein induces endothelial dysfunction by downregulating ACE2 expression, leading to attenuate the protective effect of ACE2 as the anti‐inflammatory pathway to maintain cellular hemostasis.^[^ ^6^ ^]^ Downregulation in ACE2 expression increases vascular permeability, activates the coagulation pathway, and reduces fibrinolysis (**Figure** **1**a).^[^ ^7^ ^]^ As a consequence, elevated serum inflammatory cytokines, including Interleukins (IL)‐6, IL‐1, tumor necrosis factor (TNF)‐*α*, and endothelial biomarkers, including von Willebrand factor (vWF), prime for inflammatory responses, and microvascular thrombosis.^[^ ^8^, ^9^, ^10^ ^]^ In addition to the elevated circulating cytokine levels, also known as a cytokine storm, vascular endothelialitis develops, accompanied by leukocyte recruitment and complement activation.^[^ ^11^, ^12^ ^]^ In the microcirculation, SARS‐CoV‐2 and the S protein directly enhance platelet activation and fibrin aggregation, predisposing 30–50% of COVID‐19 patients to develop thrombotic events.^[^ ^13^ ^]^

The common therapeutic targets to limit viral transmission, such as immune responses to the S protein, rely on inhibiting viral internalization by using the SARS‐CoV‐2 Spike sequence or protein to stimulate immune response in the host; thus, negating viral RNA replication and transcription in the host cells.^[^ ^14^, ^15^ ^]^ For this reason, inhibition of the Spike binding to the hACE2 receptor is the primary target of numerous vaccines and antivirals. However, the delicate regulation of the renin‐angiotensin physiological axis has renewed questions in the face of vaccine‐associated inflammation and thrombosis (Figure 1b).^[^ ^16^ ^]^

Recently, the rise in S protein mutations (e.g., Alpha variant also known as B1.1.7) identified in the United Kingdom, and the rapid spread of variants, including B.1.3510 and B.1.617.2 (also known as Delta variant first identified in India)^[^ ^17^, ^18^ ^]^ have met with an increased S protein affinity to hACE2 receptor or viral transmission that may evade the vaccine‐mediated neutralizing antibodies.^[^ ^19^ ^]^ To this end, there is an urgent clinical need to rapidly identify the patients at risk for microvascular thrombosis (blood clots) upon exposure to the SARS‐CoV‐2 variants or Spike protein‐based vaccines in a patient blood‐specific manner.

In this study, we developed a novel viral‐free microfluidic platform in which we recapitulated blood coagulation with patient‐specific whole blood and the custom‐designed nanoliposomes (diameter = 100 nm) biotinylated with the SARS‐CoV‐2 S protein subunit, S_1_ (Lipo‐S). At a shear stress of 25 dyne cm^−2^,^[^ ^20^, ^21^ ^]^ we visualized and quantified the inflammatory cytokine‐mediated microthrombosis^[^ ^22^, ^23^ ^]^ upon exposure to the most common Spike mutation D614G (Asp^614^→Gly substitution in S1 subunit) using a viral vector, and we corroborated blood coagulation using the live SARS‐CoV‐2 virus. We performed countermeasure to reverse Spike‐, and Spike mutation D614G‐mediated blood coagulation by conjugating liposomes with human‐ACE2 (Lipo‐hACE2) as a competitive S protein decoy. We used antibody anti‐IL‐6 to demonstrate the role of inflammatory cytokines to promote thrombosis. Overall, we integrated nanoliposomes and human whole blood in the microfluidic platform to rapidly identify and inhibit patient blood‐specific coagulation in response to Spike mutation; thereby, obviating the use of live SARS‐CoV‐2 to screen and prevent viral variants‐ and vaccination‐associated microthrombosis (Figure 1).

## 2 Experimental Section

### 2.1 Construction of Custom‐Designed Nanoliposomes

Construction of nanoliposomes (diameter = 100 nm) with S1 Spike proteins biotinylated to an external lipid bilayer (Lipo‐S) was previously reported.^[^ ^24^ ^]^ In brief, biotinylated cap liposomes with encapsulated rhodamine (IMF‐3951, Encapsula Nanoscience) were mixed for 1 h at room temperature with neutravidin proteins in a 1:10 ratio. After incubation at 4 °C overnight, the liposomes covalently bound with neutravidin proteins were dialyzed (300K molecular weight cut off (MWCO), Spectrum Labs) in 1X phosphate buffered saline (PBS) twice for 8 h at 4 °C. Biotinylated SARS‐CoV‐2 S1 protein (S1N‐C82E8, Acrobiosystems) were introduced to the liposome‐neutravidin mix in a ratio of 1:2 and incubated for 1 h at room temperature and at 4 °C overnight. Next, the solution was dialyzed to remove the unbound S protein and the remaining purified nanoliposome (Lipo‐S) was ready to be exposed to cells. Liposomes with hACE2 (Lipo‐hACE2) were made in a similar fashion. Rather than the SARS‐CoV‐2 S1 protein, the hACE2 protein was used to covalently bind to the liposome‐neutravidin mix and incubated for 1 h at room temperature and at 4 °C overnight. After dialysis to remove the unbound hACE2, the purified nanoliposome was ready to be used as a decoy to mitigate S protein binding to endothelial ACE2.

To confirm that the ACE2 proteins were bound to the liposomes, enzyme‐linked immunosorbent assay (ELISAs) (Human ACE2 ELISA Kit (ab235649) were performed to verify ACE2 proteins through absorbance measurements at 405 nm. The rhodamine encapsulated in liposomes could be comeasured as a fluorescent moiety (Figure S2, Supporting Information).

### 2.2 Construction of pLenti‐CMV‐SARS‐CoV‐2‐S mutation D614G (Asp 17 614→Gly) and pLenti‐CMV‐MCS‐hACE2‐IRES‐sfGFP‐SV‐Puro Vectors

The mature polypeptide of human ACE2 (GenBank NM_021804.3) was cloned in to the XbaI‐BamHI site of pLenti‐CMV‐MCS‐green fluorescent protein (GFP)‐stomatitis virus (SV)‐puro (Addgene #73582). IRES linker was previously inserted into pcDNA3‐sACE2v4‐sfGFP via BamHI restriction enzyme site. The pcDNA3‐sACE2v4‐IRES‐sfGFP insert was removed by restriction enzymes using NheI‐XhoI. Next, the NheI‐XhoI sites were converted into XbaI‐BamHI by polymerase chain reaction (PCR) (Primers: Forward‐TAGCCTAGAGCCACCATGTCAAGCT, Reverse‐CACCTGATCCCATTTGTAG AGCTCATCCATGCCATG) to be compatible with the pLenti‐CMV‐MCS‐SV40‐PURO vector. Similarly, pcDNA3.1 SARS‐CoV‐2 S D614G (Addgene‐158075) was cloned into pLenti‐CMV‐MCS‐GFP‐SV‐puro. The GFP sequence was removed by restriction enzyme digestions (XbaI‐BamHI) and replaced with SARS‐CoV‐2 S D614G insertion sequence. Previous ligase reaction the NheI and EcoRI digestion sites in SARS‐CoV‐2‐S D614G insert were converted in the XbaI‐BamHI compatible with the backbone vector. Amplicon size was evaluated through gel electrophoresis in agarose 1.2%. DNA band was excised from the gel and purified using gel clean up reaction (Macherey Nagel). DNA insert and linear destination vector were mixed at a 1:1 ratio, respectively. T4 was used for the ligase reaction and incubated overnight at 16 °C. Successful ligation of the inserted vector was verified through gel electrophoresis in agarose 2%. The DNA band was excised from the gel and purified using gel clean up reaction (Macherey Nagel).

### 2.3 Lentivirus production in HEK293T Cells

Expression clone DNA (625 ng) was mixed with packaging plasmids pVSV‐G and pCMV delta R8.2 at the ratio of 8:1 by mass. Viafect transfection reagent (Promega) was incubated with optimal‐minimal essential medium (OPTIMEM) (Gibco) (1: 12.5, v/v) for 20–30 min at room temperature, prior to adding to the DNA mix. HEK293T cells were plated until 70–90% confluence prior to transfection. After 16 h, the medium was replaced with fresh medium. After 48 h, the virus was harvested and filtered through a 0.45 µm pore filter. Lenti‐X concentrator (Takara) was mixed with the medium containing the virus in the ratio of 1:3 media:Lenti‐X. The mix was left overnight at 4 °C following a 45 min centrifugation at 1500 x *g*. The supernatant was removed by aspiration and the pellet was resuspended in 400 µL of 1 X PBS (Gibco). 

### 2.4 Screening of Lentivirus Overexpression

Human aortic endothelial cells (HAECs, S305, Cell Applications Inc) were plated at (2 × 10^5^) per well (six‐well plate) in complete medium on Day 0 and transduced on Day 1. Then, 350 µL per well of medium containing lentivector was used. The plate was rocked every 15 min for 1 h. Next, another 650 µL was added per well and left overnight. On Day 2, the medium was changed with fresh media for 48 h. Lentiviral titration was conducted by using long‐terminal‐repeat ‐ woodchuck hepatitis virus post‐transcriptional regulatory element (LTR‐WPRE) RNA quantification as previously described by Geraerts et al.^[^ ^25^ ^]^

### 2.5 SARS‐CoV‐2 Infection Studies in Biosafety Level 3 Laboratory

SARS‐CoV‐2, isolate USA‐WA1/2020, was acquired from the Biodefense and Emerging Infections (BEI) Resources of the National Institute of Allergy and Infectious Diseases. SARS‐CoV‐2 was passaged once in Vero‐E6 cells American Type Culture Collection (ATCC) and viral stocks were aliquoted and stored at −80 °C. Virus titer was determined by plaque assay using Vero E6 Cells. 0.05_MOI_ of SARS‐CoV‐2 was used for the experiments. Studies involving live SARS‐CoV‐2 virus were approved by the University of California, Los Angeles Institutional Biosafety Committee and were performed in compliance with approved BSL3 standard operating procedures. The University California Los Angeles (UCLA) BSL3 laboratory was designed in compliance with the guidelines recommended by the Biosafety in Microbiological and Biomedical Laboratories, the U.S. Department of Health and Human Services, the Los Angeles Department of Public Health, and the Center for Disease Control and Prevention.

### 2.6 Fabrication of Microfluidic Channels

Microfluidic devices were fabricated with standard polydimethylsiloxane (PDMS) molding processes. Briefly, standard lithographic techniques were used to produce a mold from a silicon master wafer spin‐coated with SU‐8 2100 (Microchem). PDMS (Sylgard 184 Elastomer Kit, Dow Corning Corporation) with a crosslinker to polymer ratio of 1:10 was used to produce the chips from the mold. PDMS and glass were activated by O_2_ plasma (reactive ion etcher series 800, 500 mTorr, 80 Watts, 30 sec) before being bonded together. The device was composed of 2 cm long straight channels (*W*_C_ = 400 µm, *H* = 100 mm). 

Channels were then treated with 1% (3‐aminopropyl)‐trimethoxysilane (Sigma‐Aldrich) in Novec 7500 Engineered Fluid (3M) for 10 min, then rinsed with 70% ethanol and baked in an oven at 65 °C for 2 h. To prepare for cell seeding, each channel was coated with 1% gelatin overnight at 4 °C. Then, channels were rinsed with Endothelial Growth Medium (EGM‐2, Lonza) and HAECs were seeded inside at a concentration of (2.0 ± 0.5) × 10^6^ mL^−1^ and ≈5700 cells remained in the channel after nonattached cells are rinsed away after 12 h.

### 2.7 SARS‐CoV‐2 in a Microfluidic Platform

To mimic the endothelial response to the SARS‐CoV‐2 infection, the microfluidic channels were seeded with HAECs and they are exposed to the SARS‐CoV‐2 virus. SARS‐CoV‐2 at a concentration of 0.05_MOI_ was introduced to the endothelialized channels in the BSL3 laboratory in compliance with standard BSL3 operating procedures and it was incubated for 16 h at 37 °C.

The S protein‐mediated effects were compared on thrombosis in two additional conditions: 1) Lipo‐S and 2) a lentivirus with the Spike mutation D614G (Asp^614^→Gly substitution in S1 subunit). HAECs were also exposed to liposomes enriched with SARS‐CoV‐2 Spike protein (Lipo‐S), the liposome preparations were diluted to 1:1000 in EGM‐2 with a working concentration of 19.65 µg mL^−1^, followed by introduction into the microfluidic channel for 16 h incubation a 37 °C. Three conditions were studied: a negative control without any liposomes, a liposome control (Lipo‐CTRL) without any S protein, and the Lipo‐S. After 16 h, the liposome preparation was rinsed with EGM‐2 prior to the coagulation experiment. Furthermore, the effect of Spike variant was evaluated and a viral vector expressing D614G Spike mutation was also used for the purpose of this study (Lenti‐S D614G). A lentivirus control (Lenti‐CTRL) was also introduced into a separate channel for 16 h. Both Lenti‐CTRL and Lenti‐S D614G were used at 15 multiplicity of infection (MOI). In addition, with the purpose of reducing the thrombotic effect led by the aforementioned conditions, Lipo‐ACE2 and anti‐IL6 antibodies were used in combination with SARS‐CoV‐2, Lipo‐S, and Lenti‐S D614G. Next, the channel media was replaced with fresh EGM‐2. A total of four replicates were completed for each condition. 

### 2.8 Blood Coagulation Experiments and Image Analysis

Human whole blood was collected into 3.2% sodium citrate tubes and used within 2 h of blood draw to avoid preactivation of platelets.^[^ ^24^ ^]^ All samples were collected from healthy adults not using antiplatelet or anticoagulant therapy, upon consent and under approval from the Institutional Review Board. The setup of the coagulation experiment consisted of a syringe pump pulling 550 µL of blood through each channel at 30 µL min^−1^, producing a shear rate of around 750 s^–1^. After 1 min of blood flow, 100 × 10^−3^ m calcium chloride (CaCl_2_) was added to the blood reservoir in a 1:10 ratio to restore coagulation.^[^ ^26^, ^27^, ^28^ ^]^

After 15 min of blood flow, 1X PBS was used to rinse the channels of any blood that was not clotted and then 4% paraformaldehyde was perfused into the channels to fix the cells and clot for 20 min at room temperature. The fixative was flushed out with 1X PBS and the channels were prepared for antibody staining. Antibodies against platelet CD‐61 (44‐876, ThermoFisher), and fibrin (F9902, Sigma Aldrich), were perfused into the individual microfluidic channels, incubated overnight at 4 °C, followed by washing, and labeling with goat‐antimouse (SAB4600335, Millipore Sigma) or antirabbit (A‐21428, ThermoFisher) fluorescently labeled IgGs for visualization. Inverted fluorescence microscope (Nikon Eclipse Ti) was used to obtain images from the center region of interest of each channel (3.5 mm long by 400 µm wide) to avoid entrance and outlet flow effects on clot formation. The image stack was analyzed in ImageJ by obtaining the maximum intensity image, thresholding using the Moments method to compute a binary image and quantifying the number of white pixels representing platelet aggregation or fibrin deposition.^[^ ^29^ ^]^

### 2.9 Computational Analysis of Microcirculatory Blood Flow and Shear Stress

COMSOL Multiphysics v5.3 (COMSOL Inc, Burlington, MA) finite element code was used to simulate the 3D flow in the microfluidic channel. The blood was assumed as incompressible non‐Newtonian fluid and was modeled using the power‐law equation. A fully developed flow boundary condition was applied at the inlet and zero‐pressure boundary condition at the outlet to solve the steady‐state Navier–Stokes equations. The simulations determined that a flow rate of 30 µL min^−1^ enacted the desired physiological wall shear stress of 25 dyne cm^−2^ in the system.

### 2.10 RNA Extraction and Reverse Transcription‐Polymerase Chain Reaction (RT‐PCR)

Total RNA from transfected HAECs was extracted using the Quick RNA Miniprep (Zymo Research) according to the manufacturer's protocol. 250 ng RNA were reverse transcribed into cDNA with random primer by 5x All‐In‐One RT Mastermix (abm). 2 ng cDNA product (relative to RNA amount) was amplified by standard PCR with Taq DNA polymerase (Bright Green, No‐ROX Kit, abm) and primers. 

Primer sequences were designed using National Center for Biotechnology Information (NCBI) software (**Table** **1**). Glyceraldehyde 3‐phosphate dehydrogenase (GAPDH) RNA was used as the housekeeping control for each gene and the target gene was amplified in duplex in PCR mixtures (10 µL final volume) containing 5 µL Sybr Green PCR Master Mix (abm), 1 µL cDNA template, 2 µL optimized primers and 3 µL of nuclease‐free water. PCR thermal cycle parameters were as follows: 95 °C for 5 min, 62 °C for 30 s, and 72 °C for 30 s for 40 cycles. Reactions were performed and fluorescence was monitored in a Real time‐PCR System, CFX Connect (Biorad). Relative expression level was defined as the ratio of target gene mRNA level to GAPDH mRNA expression using the delta‐delta Ct method. 

**Table 1:** Primer Sequences used for RT‐PCR

Gene name	Forward primer sequence	Reverse primer sequence
*ADAMTS13*	ACAGCCAACCTCACCTCGTC	CCAGGATCCGTGTCGTCCTC
*CXCR4*	GGAAATGGGCTCAGGGGACT	TGGTGGGCAGGAAGATTTTATTGAA
*FGA*	ATGGCAGATGAGGCCGGAAG	TTACTGGATCCCGGTAGCTTGA
*FURIN*	AGGTGCGGAAGACCGTGAC	CTGCGGAGTAGTCATGTGGC
*HMOX1*	TGCACACCCAGGCAGAGAAT	CTTGAAGCCGTCTCGGGTCA
*HRG*	TCGACCCTCAGGAACATGAGAAC	GAAGAACGCTCATGCCCACC
*IL1α*	ACAAGTGGTGTTCTCCATGTCC	TGGGATCTACACTCTCCAGC
*IL15*	TTGGGAACCATAGATTTGTGCAG	GGGTGAACATCACTTTCCGTAT
*IL6*	GGTACATCCTCGACGGCATCT	GTGCCTCTTTGCTGCTTTCAC
*IL8*	ACTTCCAAGCTGGCCGTGG	TGGCAAAACTGCACCTTCACA
*ITGA2B*	CGAAACTGGCCGAAGTGGG	TTGTAGCCATCCCGGTCGAG
*MCP1*	TGTCCCAAAGAAGCTGTGATCT	GGAATCCTGAACCCACTTCTG
*P‐SEL*	GGACCGGAAGTGGTGCAATG	ATGGTTCCTTCACTGGGGGC
*PAI‐1*	CTTGGGAAAGGAGCCGTGGA	GGGCAGTTCCAGGATGTCGT
*PIGA*	CACCGTGCCTCAGCTACACT	CAGGCACTGAGAGAGCTGGT
*PLG*	ACCGTGCCTCAGCTACACTC	AATGTGGCTTTCCACGCCTC
*SERPINE*	GGACCGCAACGTGGTTTTCT	GGGCCATGCCCTTGTCATCA
*TLR1*	TCTGTTTTTGTGGCCAGGGTC	TCCTTTTGTAGGGGTGCCCA
*TLR2*	CGGAGTTCTCCCAGTGTTTGGT	GCAGGAGGGGTGTTGGAAACT

### 2.11 Viral RNA Extraction and Quantitative Real‐Time RT‐PCR (qRT‐PCR)

One hundred microliter cell culture supernatant was harvested for viral RNA extraction using Quick‐RNA Microprep Kit (R1050) according to the manufacturer's instructions. RNA was eluted in 10 µL RNase‐free water. Reverse transcription was performed with a 5x All‐In‐One RT Mastermix (abm) and qRT‐PCR was performed on BioRad Real‐time PCR system (BioRad) with TB BrightGreen Premix Taq II (abm). cDNA was synthesized in 20 µL reaction and 2 µL cDNA was used as a template for quantitative PCR. The primers used for quantitative PCR were RBD Forward: 5’‐CAATGGTTTAACAGGCACAGG‐3’ and RBD‐Reverse:5’‐CTCAAGTGTCTGTGGATCACG‐3. PCR amplification was performed as follows: 95 °C for 5 min followed by 40 cycles consisting of 95 °C for 15 s, 54 °C for 15 s, 72 °C for 30 s. The dose‐response curves were plotted from viral RNA copies versus the drug concentrations using GraphPad Prism 6 software.

### 2.12 Statistical Analysis

All data are presented as mean ± standard error of the mean (SEM). The PCR results consisted of three independent experiments and all microfluidic channel studies consisted of four independent experiments for each condition. A statistical one‐way analysis of variance (ANOVA) test was used to evaluate three or more groups, and a two‐way ANOVA was used to evaluate three or more groups with different conditions. Dunnett's post hoc analysis was conducted to compare differences between the liposome control and the Lipo‐S, in the presence or absence of Lipo‐hACE2, as well as for the lentivirus control to the Lenti‐S, in the presence or absence of Lipo‐hACE2. *P*‐values < 0.05 were statistically significant and GraphPad Prism software was used for statistical analysis (GraphPad Software, La Jolla, CA).

## 3 Results

### 3.1 Microfluidic Channels with Lipo‐Spike Recapitulate SARS‐CoV‐2‐Mediated Immune and Thrombotic Response

**Figure 2:**
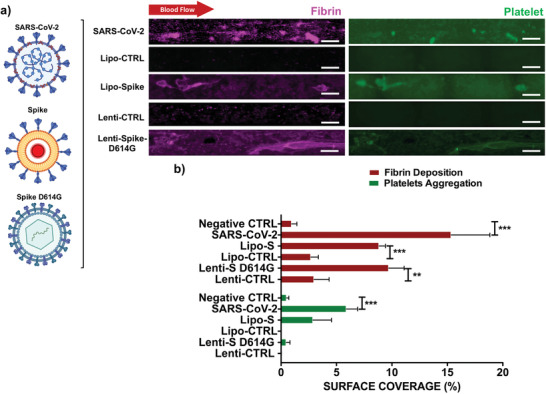
SARS‐CoV‐2 and Spike‐mediated thrombotic effect in simulated microcirculation. a) Representative images of fibrin deposition (in magenta) and platelet‐platelet aggregates (in green) in the HAEC‐seeded microfluidic channels were compared with exposure to the live SARS‐CoV‐2 virus, the Spike proteins, and the Spike variant D614G via viral vector (scale bar = 100 µm). Fibrin nodules were observed as dense magenta aggregates in the center of the channels in the presence of SARS‐CoV‐2 and also confirmed in response to Lenti‐Spike mutation D614G (*n* = 4). b) Bar graphs demonstrate a significant increase in surface coverage of platelet aggregation in response to the SARS‐CoV‐2 (***p < 0.001 vs noninfected cells, *n* = 4). Fibrin deposition was also significant in response to Spike and the Spike mutation D614G. One‐way ANOVA and post hoc Dunnett's test.

Whole blood from healthy participants was introduced in the endothelialized microfluidic channels in the presence of the conditions (Figure 1c) at a Reynolds number of 0.61 and fluid wall shear stress of 25 dyne cm^−2^ (Figure S1, Supporting Information). After 15 min, platelet aggregation and fibrin deposition were quantified (**Figure** **2**a,b). These fibrin depositions were elongated in the direction of blood flow and the thrombus formation tended to exhibit as a core of platelets (green) surrounded by a layer of fibrin (magenta). Fibrin deposition was observed in response to SARS‐CoV‐2 infection in HAECs which exhibited the highest percentage of surface coverage in the microfluidic channel. Fibrin deposition was also prominent throughout the entire length of the microfluidic channels with surface coverage of 17% ± 5.3% (***p < 0.001 vs control, *n* = 4) and platelet aggregation of 6.91% ± 0.53% (**p < 0.001 vs control, *n* = 4). Fibrin surface coverage for Lipo‐S was 9.53% ± 1.46% (**p < 0.01 vs control, *n* = 4), similarly to the Lenti‐S variant D614G which also promoted significant fibrin deposition surface coverage at 9.56% ± 3.23% (**p < 0.01 vs control, *n* = 4) (Figure 2a,b). These results support the notion that the Spike protein is sufficient to develop hyperfibrinogenemia in the setting of COVID‐19‐mediated coagulopathy.^[^ ^30^ ^]^

### 3.2 SARS‐CoV‐2 and Sub‐Genomic Sequences‐Mediated Cytokine Expression

**Figure 3:**
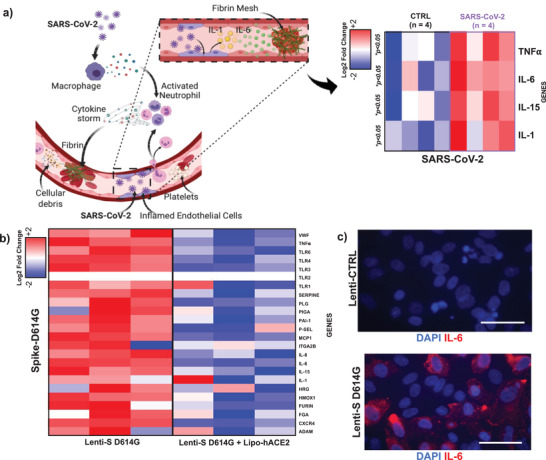
SARS‐CoV‐2 and Spike‐mediated inflammatory cytokines regulates coagulation cascade. a) Tissue factor (TF) binds to coagulation factor VII (FVII) to initiate the thrombosis. SARS‐CoV‐2 infection also induces endothelial release of cytokines (such as IL‐1, IL‐6, and TNF‐*α*) that mediate platelet activation and coagulation cascades. SARS‐COV‐2 treatment for 48 h significantly upregulated HAEC mRNA expression of TNF‐*α*, IL‐1, IL‐6, and IL‐15 as demonstrated by the heatmap (*p < 0.05 CTRL vs SARS‐COV‐2, *n* = 4 by qRT‐PCR). The heat map was constructed by using Euclidean distance with average linkage. The *Z*‐score centered log2‐transformed gene in each sample is presented by a color scale, and gene upregulation is denoted in blue, and downregulation in red. b) Spike mutation D614G inflammatory effect was tested HAECs. A microarray heatmap represents 22 genes and selected control genes in HAECs in response to Spike D614G. Hierarchical clustering heatmap reveal differentially expressed genes in response to Lenti‐S mutation (in the presence or absence of Lipo‐hACE2), normalized to the lenti‐CTRL respectively. The heatmap was constructed as previously described, and the Z‐score centered log2‐transformed gene in each sample was presented as a color scale. Each condition was performed in triplicate (*n* = 3). In addition to increased cytokines and chemokines mRNA expression level, higher mRNA expression level of endothelial marker of thrombosis, such as vWF and PAI‐1, was also observed. Furthermore, the activation of the Toll‐like receptor signaling pathway suggests its crucial role in enhancing downstream inflammation and thrombosis (see Figure S5, Supporting Information) (*p < 0.05: *n* = 3). c) Immunocytochemical analysis showed Lenti‐S D614G increasing protein level of IL‐6 (in red). Nuclei were stained with DAPI (scale bar: 50 µm).

SARS‐CoV‐2 infection induces a release of endothelial cytokines, including IL‐1*α*, IL‐6, TNF*α*, to promote platelet activation and the coagulation cascade (Figure 2a,b). SARS‐CoV‐2 (0.05_MOI_) significantly elevated mRNA expression of TNF‐*α*, IL‐1*α*, IL‐6, and IL‐15 (*p < 0.05 vs noninfected HAECs, *n* = 3) (**Figure** **3**a and Figure S4, Supporting Information). We further quantified cytokine gene expression in response to Lenti‐S D614G. Lenti‐S D614G exposure upregulated cytokines (e.g., TNF‐*α*, IL‐6, and IL‐15) and chemokines (e.g., MCP1), together with aberrantly elevated level of endothelial markers, such as vWF (*p < 0.05 vs control, *n* = 3) potentially through overexpression of Toll‐like receptors (Figure 3b). In addition, we demonstrated that Spike‐mediated inflammatory mRNA expression was reduced by exposing HAECs to Lipo‐hACE2 together with Lenti‐S, suggesting that enrichment of exogenous ACE2 might be potentially responsible for inhibiting SARS‐CoV‐2 inflammation and thrombus formation (Figure 3b).

Furthermore, anti‐IL‐6 staining was prominent in Lenti‐S D614G transduced HAECs (Figure 3c). These findings further support IL‐6 as a therapeutic target to mitigate SARS‐CoV‐2‐mediated microvascular thrombosis.

In addition to increased cytokines and chemokines mRNA expression level, higher mRNA expression level of endothelial marker of thrombosis, such as vWF and plasminogen activator inhibitor‐1 (PAI‐1), was also observed. Furthermore, the activation of the Toll‐like receptor signaling pathway suggests its crucial role in enhancing downstream inflammation and thrombosis (see Figure S5, Supporting Information) (*p < 0.05: *n* = 3). c) Immunocytochemical analysis showed Lenti‐S D614G increasing protein level of IL‐6 (in red). Nuclei were stained with 4',6‐diamidino‐2‐phenylindole (DAPI) (scale bar: 50 µm).

### 3.3 Lipo‐hACE2 and Anti‐IL‐6 Attenuate SARS‐CoV‐2‐Mediated Inflammation and Thrombosis

**Figure 4:**
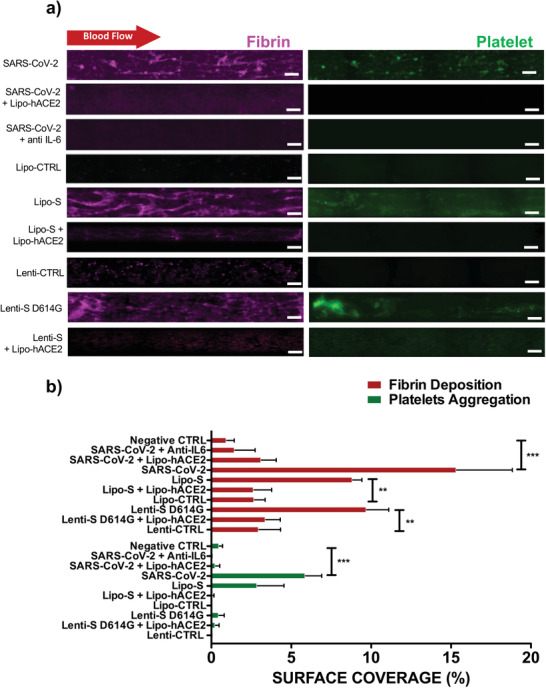
Lipo‐hACE2 and anti‐IL‐6 attenuate SARS‐CoV‐2‐mediated inflammation and thrombosis. a) In the endothelialized microfluidic platform, HAECs were exposed to SARS‐CoV‐2, Lenti‐S D614G, or Lipo‐S in the presence or absence of Lipo‐hACE2 or anti‐IL‐6 (scale bar = 100 µm). Lipo‐hACE2 and anti‐IL‐6 attenuated SARS‐CoV‐2, Lenti‐S, or Lipo‐S‐mediated fibrin deposition. b) Both Lipo‐hACE2 and anti‐IL‐6 reduced SARS‐CoV‐2, Lipo‐S, and Lenti‐S mediated increases in surface coverage of fibrin and platelet deposition (*p < 0.05 vs Lipo‐S or Lenti‐S mutation; ***p < 0.001 vs SARS‐CoV‐2, *n* = 4).

We demonstrated Lipo‐hACE2 and IL‐6 as two target countermeasures. In microfluidic channels, live SARS‐CoV‐2 virus‐, Lipo‐S‐, and Lenti‐S‐mediated inflammation and thrombosis were significantly reduced by either Lipo‐hACE2 or an anti‐IL‐6 antibody. In our study we observed that IL‐6 increase expression due to SARS‐CoV‐2 or Spike exposure (Figure 2a,b) triggered blood coagulation in the simulated microfluidic platform and its effect is abrogated by using antibody anti‐IL‐6 Similarly, the addition of Lipo‐hACE2 reduced fibrin coverage in the presence of the SARS‐CoV‐2 live virus by 6.8% ± 2.2%, in the presence of Lipo‐S and Lenti‐S by 20.1% ± 2.5% (*p < 0.05 vs control, *n* = 4) and 6.44% ± 1.7%, respectively (*p < 0.05 vs control, *n* = 4). Lipo‐hACE2 further reduced platelet surface coverage in the presence of Lenti‐S by 9.3% ± 1.5% (*p < 0.05 vs control, *n* = 4) (**Figure** **4**a,b).

Furthermore, at an effective concentration of 2.13 µg mL^−1^, Lipo‐hACE2 inhibited SARS‐CoV‐2 replication, and at 0.85 µg mL^−1^ Lipo‐hACE2 inhibited SARS‐CoV‐2‐mediated cell death (Figure S3, Supporting Information). Taken together, Spike protein induced‐microthrombosis can be abrogated by the use of Lipo‐hACE2 or Anti‐IL‐6 antibodies.

### 3.4 Heatmaps and Interactome Analyses to Relate Inflammation with Coagulation

**Figure 5:**
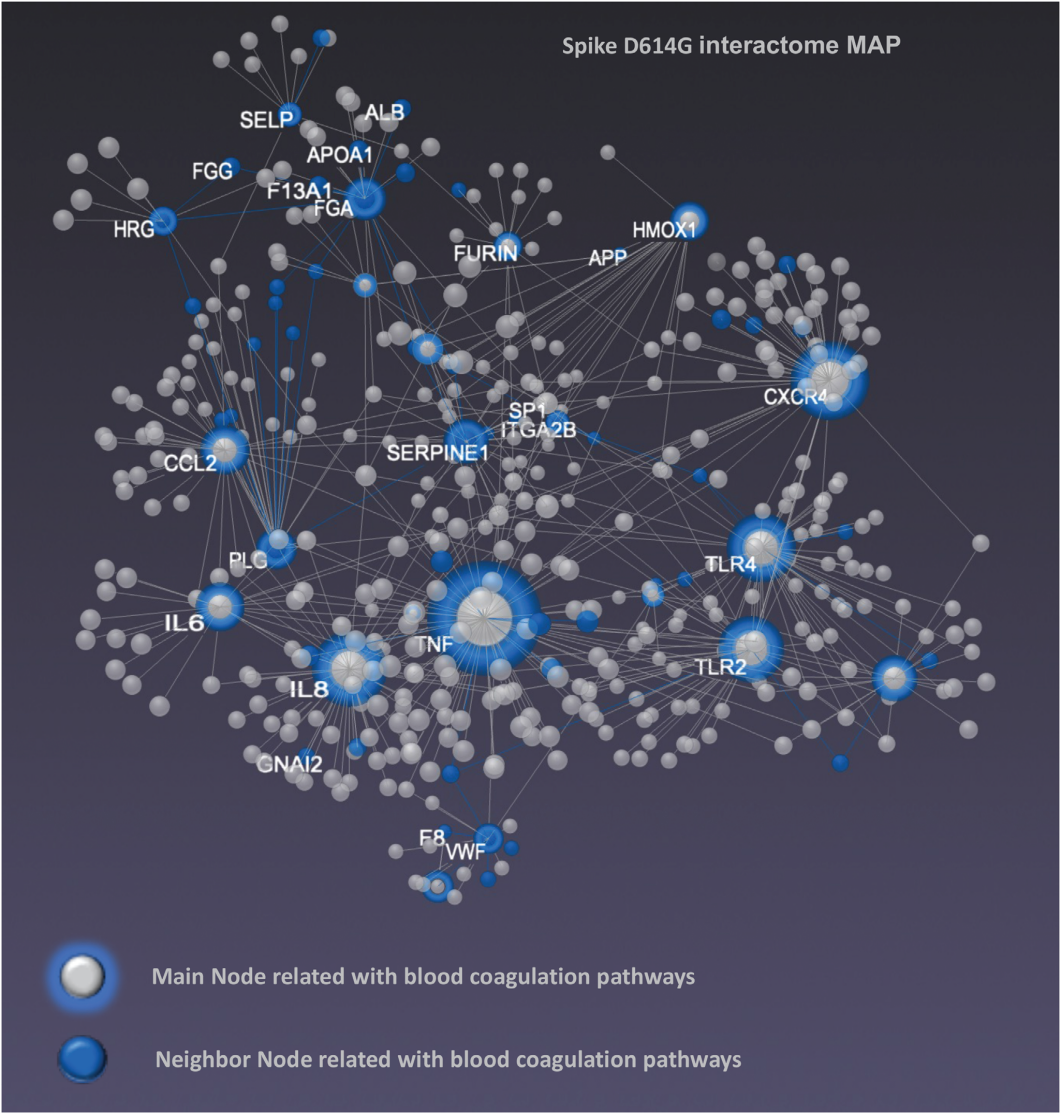
Network visualization with the central panel depicts a force‐directed gene‐gene network in 3D space. Graph clusters from OmicsNET reveal unique difference networks between the intersection of COVID‐19 disease‐type constructed networks and each type‐specific network with the genes shared by Spike. KEGG pathway analysis was performed for the multiomics layered network (mRNA, neighboring genes, OmicsNet). We revealed that the majority of the overrepresented biological processes following SARS‐CoV‐2 infection were regulated by the Toll‐like receptor signaling, inflammasome activation, intrinsic coagulation pathway, and signaling by Interleukins. COVID‐19‐induced cytokine storm leads to systemic complications and multiorgan damage (see Figure S5 and Table S1, Supporting Information). Blue/gray main nodes and blue neighbor nodes show gene involved in blood coagulation signaling pathways.

To interconnect specific gene clusters underlying inflammation and coagulation, we used log2‐fold change from qPCR analysis, and we performed multiomic data integration using the web‐based tool OmicsNet (**Figure** **5**).^[^ ^31^ ^]^ Genes were uploaded based on corresponding Entrez gene ID. If the network exceeded 3000 nodes, the minimum network setting was taken into account by the OmicsNet algorithm that identified the smallest subnetwork and only interactors that targeted the seeded nodes were considered. Functional exploration of the network analysis was conducted by the OmicsNet software that applied hypergeometric tests and the built‐in knowledgebase gene sets from Kyoto Encyclopedia of Genes and Genomes (KEGG) and Reactome (Table S1, Supporting Information). qPCR data (Figure S5, Supporting Information) were presented as mean ± standard deviation (SD) and statistical significance was calculated using one‐way ANOVA. A *p*‐value < 0.05 was considered statistically significant. KEGG pathway analysis was performed for the multiomics layered network (mRNA, neighboring genes, OmicsNet). We revealed that the majority of the overrepresented biological processes following SARS‐CoV‐2 infection were regulated by the Toll‐like receptor signaling, inflammasome activation, intrinsic coagulation pathway, and signaling by interleukins. Consistently, COVID‐19‐induced cytokine storm leads to systemic complications and multiorgan damage.^[^ ^32^ ^]^

## 4 Discussion

In this study, we integrated nanoliposomes, viral vectors, and human whole blood in the microfluidic platform to recapitulate blood coagulation upon exposure to SARS‐CoV‐2 or Spike mutation (D614G). In the face of rising Spike mutations and spread of viral variants, vascular thrombosis has been recognized as contributing to increased mortality in COVID‐19 patients,^[^ ^33^ ^]^ and emerging cases of apparent secondary immune thrombosis after SARS‐CoV‐2 vaccination have been reported and reached public health issue.^[^ ^34^ ^]^ Despite different vehicles, all the SARS‐CoV‐2 vaccinations share the use of Spike sequence (RNA/DNA) or protein for host immune response stimulation.^[^ ^35^ ^]^

As a proof‐of‐concept study we showed that our novel microfluid platform is the ideal tool for an early detection of thrombosis in the event of SARS‐CoV‐2 infection or following Spike‐related vaccination therapies. Using patient‐specific whole blood we are able to develop a precision type medicine diagnostic tool which creates customization of healthcare, and it can serve as a testing platform for medical decisions, treatments, practices or products that can be tailored to patients, instead of a one‐drug‐fits‐all model.

To this end, we recapitulated a microvascular environment to streamline the testing of the Spike protein and a variant (Asp^614^→Gly substitute in S1) of SARS‐CoV‐2 in our integrated platform for individualized prediction of microthrombosis. Spike microvascular thrombosis has not been well elucidated in human microcirculation and there remains a paucity of studies related to the signaling pathways underlying Spike‐mediated blood coagulation. For the purpose of this study, we developed a fluorescently tractable nanoparticle system in which the binding between nanoparticles Spike proteins‐conjugated and the ACE2 in human aortic endothelium promoted blood coagulation in the presence of human whole blood.

Furthermore, we evaluated the role of the Spike mutation D614G using viral vectors and we validated our results with the use of live SARS‐CoV‐2 virus to demonstrate platelet aggregation and fibrin deposition on the endothelium as the quantifiable phenotypes for thrombosis progression at a shear stress of 25 dyne cm^−2^ which simulates laminar shear stress on straight portions of the vasculature.^[^ ^20^, ^27^, ^36^, ^37^ ^]^ These areas have been shown by our group and others to express higher levels of ACE2 in a flow dependent manner.^[^ ^24^, ^36^, ^38^ ^]^ Fibrin deposition is well known as an indication of infection, the production of fibrin matrices is an innate physiological response to delay the spread of a pathogen in the blood stream and is a part of the healthy immune response.^[^ ^39^, ^40^ ^]^

We validated the Spike protein binding to hACE2 of vascular endothelium (data not shown) underlying the disruption of the Renin‐Angiotensin System (RAS) system,^[^ ^41^ ^]^ leading to the activation of inflammatory pathways and thrombus formation.^[^ ^6^, ^9^, ^10^, ^35^, ^41^ ^]^ We demonstrated that the presence of Spike D614G is sufficient to recapitulate SARS‐CoV‐2‐mediated inflammation by upregulating cytokine expression (e.g., IL‐1, IL‐6, IL‐15, and TNF‐*α*), and chemokines (e.g., MCP1) together with aberrantly elevated level of endothelial markers, such as von Willebrand Factor (vWF), which can potentially be the link for the significant platelet aggregation and fibrin deposition through Toll‐like receptor signaling pathway. Similar outcome in terms of blood coagulation was also observed in the channels exposed to Spike protein conjugated with nanoparticles,^[^ ^18^ ^]^ suggesting that despite the different vehicles the presence of the spike proteins is sufficient to stimulate thrombosis.

This suggests that the interaction between the Spike proteins and the ACE2 receptors led to a shift in ACE2 catalytic equilibrium, inhibiting the concentrations of substrates such as angiotensin II, dynorphin‐13, apelin‐13 and products such as angiotensin (1–7) and (1–9), dynorphin‐12 and apelin‐12. Ultimately, accumulation of these substrates primes for uncontrolled inflammation which is observed in COVID‐19 patients. Thus, the inflammasome activation promoted increased level of IL‐6 via IL‐1,^[^ ^42^ ^]^ leading to thrombosis.^[^ ^43^, ^44^, ^46^ ^]^

Circulating IL‐6 has been reported to be a strong independent marker correlated to increased mortality in several pathologies.^[^ ^46^, ^47^ ^]^ Furthermore, high IL‐6 was also linked to fatalities in COVID‐19 patients.^[^ ^48^, ^49^ ^]^ To mitigate Spike protein‐mediated thrombosis, we used antibody anti‐IL6 in the microfluidic platform to reverse the cytokine‐mediated blood coagulation. This finding is consistent with the use of IL‐6 receptor antagonists, including Tocilizumab and Sarilumab, to improve outcomes and survival in critically ill patients with COVID‐19.^[^ ^50^, ^51^, ^52^ ^]^ In addition, we created custom‐designed nanoparticles conjugated with hACE2 as a decoy for the SARS‐CoV‐2 Spike protein, and we demonstrated that Lipo‐hACE2 inhibited microthrombosis in the presence of the Spike, the D614G variant and the live SARS‐CoV‐2. Thus, our microfluidic platform recapitulated the therapeutic effects of anti‐IL‐6 and Lipo‐hACE2 to prevent blood coagulation.

## 5 Conclusions

We demonstrate the feasibility of a rapid patient‐specific diagnosis of thrombosis in response to SARS‐CoV‐2 or Spike protein mutations. This integration of nanotechnology, viral vectors, and human whole blood in a viral‐free microfluidic environment provides the basis to predict the risk associated with SARS‐CoV‐2 or Spike protein in an individualized manner. Our finding is timely to address SARS‐CoV‐2 or vaccination‐associated thrombosis. Therefore, our platform further paves the way for high‐throughput screening of large viral or chemical libraries using purified recombinant proteins or viral vectors. This integration, along with the development of nanoliposomes (Lipo‐hACE2) and selective countermeasure with anti‐IL‐6 is translational for rapid identification and prediction of thrombotic phenotypes and target countermeasures amidst the surge in Spike protein mutations and viral variants.

## Conflict of Interest

The authors declare no conflict of interest.

## Author Contributions

S.S. and A.L. contributed equally to this work. The author contribution is as follows: conceptualization (S.S., A.L., S.C., T.K.H.); methodology (S.S., A.L.); investigation (S.S., A.L., S.C., C.W.); visualization (S.S., A.L., T.K.H., J.C., M.R.); supervision (T.K.H., R.S., Z.L., N.A., D.D.C., B.J.D.); writing—original draft (S.S., A.L., S.C., A.M.B.‐M., T.K.H.); writing—review and editing (S.S., A.L., S.C., A.M.B.‐M., T.K.H.).

## Supporting information

Supporting Information

## Data Availability

The data that support the findings of this study are available from the corresponding author upon reasonable request.

## References

[1] S. Mas‐Coma , M. K. Jones , A. M. Marty , One Health 2020, 9, 100132. 32368611 10.1016/j.onehlt.2020.100132 PMC7184197

[2] E. J. Lee , D. B. Cines , T. Gernsheimer , C. Kessler , M. Michel , M. D. Tarantino , J. W. Semple , D. M. Arnold , B. Godeau , M. P. Lambert , J. B. Bussel , Am. J. Hematol. 2021, 96, 534. 33606296 10.1002/ajh.26132 PMC8014568

[3] A. Kwetkat , H. J. Heppner , Interdiscip. Top. Gerontol. Geriatr. 2020, 43, 73. 32305984 10.1159/000504491

[4] A. Remlabeevi , T. Mathew , G. S. H. Nair , G. L. Rajasekharan Nair , M. R. Alex , medRxiv 2021, 10.1101/2021.05.19.21257317

[5] Y. Huang , C. Yang , X. F. Xu , W. Xu , S. W. Liu , Acta Pharmacol. Sin. 2020, 41, 1141. 32747721 10.1038/s41401-020-0485-4 PMC7396720

[6] R. A. Fraga‐Silva , B. S. Sorg , M. Wankhede , C. Dedeugd , J. Y. Jun , M. B. Baker , Y. Li , R. K. Castellano , M. J. Katovich , M. K. Raizada , A. J. Ferreira , Mol. Med. 2010, 16, 210. 20111697 10.2119/molmed.2009.00160 PMC2811560

[7] Y. X. Gue , D. A. Gorog , Eur. Heart J. 2020, 41, 3198. 32691041 10.1093/eurheartj/ehaa534 PMC7454491

[8] C. Lucas , P. Wong , J. Klein , T. B. R. Castro , J. Silva , M. Sundaram , M. K. Ellingson , T. Mao , J. E. Oh , B. Israelow , T. Takahashi , M. Tokuyama , P. Lu , A. Venkataraman , A. Park , S. Mohanty , H. Wang , A. L. Wyllie , C. B. F. Vogels , R. Earnest , S. Lapidus , I. M. Ott , A. J. Moore , M. C. Muenker , J. B. Fournier , M. Campbell , C. D. Odio , A. Casanovas‐Massana , I. T. Yale , R. Herbst , A. C. Shaw , R. Medzhitov , W. L. Schulz , N. D. Grubaugh , C. Dela Cruz , S. Farhadian , A. I. Ko , S. B. Omer , A. Iwasaki , Nature 2020, 584, 463. 32717743 10.1038/s41586-020-2588-y PMC7477538

[9] T. Herold , V. Jurinovic , C. Arnreich , B. J. Lipworth , J. C. Hellmuth , M. von Bergwelt‐Baildon , M. Klein , T. Weinberger , J. Allergy Clin. Immunol. 2020, 146, 128. 32425269 10.1016/j.jaci.2020.05.008 PMC7233239

[10] G. Goshua , A. B. Pine , M. L. Meizlish , C. H. Chang , H. Zhang , P. Bahel , A. Baluha , N. Bar , R. D. Bona , A. J. Burns , C. S. Dela Cruz , A. Dumont , S. Halene , J. Hwa , J. Koff , H. Menninger , N. Neparidze , C. Price , J. M. Siner , C. Tormey , H. M. Rinder , H. J. Chun , A. I. Lee , Lancet Haematol. 2020, 7, e575. 32619411 10.1016/S2352-3026(20)30216-7 PMC7326446

[11] C. Magro , J. J. Mulvey , D. Berlin , G. Nuovo , S. Salvatore , J. Harp , A. Baxter‐Stoltzfus , J. Laurence , Transl. Res. 2020, 220, 1. 32299776 10.1016/j.trsl.2020.04.007 PMC7158248

[12] C. Zhang , Basic Res. Cardiol. 2008, 103, 398. 18600364 10.1007/s00395-008-0733-0 PMC2705866

[13] S. Zhang , Y. Liu , X. Wang , L. Yang , H. Li , Y. Wang , M. Liu , X. Zhao , Y. Xie , Y. Yang , S. Zhang , Z. Fan , J. Dong , Z. Yuan , Z. Ding , Y. Zhang , L. Hu , J. Hematol. Oncol. 2020, 13, 120. 32887634 10.1186/s13045-020-00954-7 PMC7471641

[14] J. ter Meulen , E. N. van den Brink , L. L. Poon , W. E. Marissen , C. S. Leung , F. Cox , C. Y. Cheung , A. Q. Bakker , J. A. Bogaards , E. van Deventer , W. Preiser , H. W. Doerr , V. T. Chow , J. de Kruif , J. S. Peiris , J. Goudsmit , PLoS Med. 2006, 3, e237. 16796401 10.1371/journal.pmed.0030237 PMC1483912

[15] L. Enjuanes , S. Zuniga , C. Castano‐Rodriguez , J. Gutierrez‐Alvarez , J. Canton , I. Sola , Adv. Virus Res. 2016, 96, 245. 27712626 10.1016/bs.aivir.2016.08.003 PMC7112271

[16] A. Greinacher , T. Thiele , T. E. Warkentin , K. Weisser , P. A. Kyrle , S. Eichinger , N. Engl. J. Med. 2021, 384, 2092. 33835769 10.1056/NEJMoa2104840 PMC8095372

[17] R. Wang , J. Chen , K. Gao , Y. Hozumi , C. Yin , G. W. Wei , Commun. Biol. 2021, 4, 228. 33589648 10.1038/s42003-021-01754-6 PMC7884689

[18] J. Ogawa , W. Zhu , N. Tonnu , O. Singer , T. Hunter , A. L. Ryan , G. M. Pao , bioRxiv 2020, 2020.2007.2021.214932.

[19] W. F. Garcia‐Beltran , E. C. Lam , K. St Denis , A. D. Nitido , Z. H. Garcia , B. M. Hauser , J. Feldman , M. N. Pavlovic , D. J. Gregory , M. C. Poznansky , A. Sigal , A. G. Schmidt , A. J. Iafrate , V. Naranbhai , A. B. Balazs , Cell 2021, 184, 2372. 33743213 10.1016/j.cell.2021.03.013 PMC7953441

[20] A. D. van der Meer , A. A. Poot , M. H. Duits , J. Feijen , I. Vermes , J. Biomed. Biotechnol. 2009, 2009, 823148. 19911076 10.1155/2009/823148 PMC2775250

[21] D. Kwasny , K. Kiilerich‐Pedersen , J. Moresco , M. Dimaki , N. Rozlosnik , W. E. Svendsen , Biomed. Microdevices 2011, 13, 899. 21739185 10.1007/s10544-011-9559-x

[22] K. B. Neeves , S. L. Diamond , Lab Chip 2008, 8, 701. 18432339 10.1039/b717824g PMC2612095

[23] R. W. Muthard , S. L. Diamond , Lab Chip 2013, 13, 1883. 23549358 10.1039/c3lc41332b PMC3660965

[24] N. Kaneko , S. Satta , Y. Komuro , S. D. Muthukrishnan , V. Kakarla , L. Guo , J. An , F. Elahi , H. I. Kornblum , D. S. Liebeskind , T. Hsiai , J. D. Hinman , Stroke 2021, 52, 260. 33161843 10.1161/STROKEAHA.120.032764 PMC7769899

[25] M. Geraerts , S. Willems , V. Baekelandt , Z. Debyser , R. Gijsbers , BMC Biotechnol. 2006, 6, 34. 16836756 10.1186/1472-6750-6-34 PMC1534021

[26] A. Jain , A. Graveline , A. Waterhouse , A. Vernet , R. Flaumenhaft , D. E. Ingber , Nat. Commun. 2016, 7, 10176. 26733371 10.1038/ncomms10176 PMC4729824

[27] A. Jain , A. D. van der Meer , A. L. Papa , R. Barrile , A. Lai , B. L. Schlechter , M. A. Otieno , C. S. Louden , G. A. Hamilton , A. D. Michelson , A. L. Frelinger 3rd , D. E. Ingber , Biomed. Microdevices 2016, 18, 73. 27464497 10.1007/s10544-016-0095-6 PMC4963439

[28] K. B. Neeves , O. J. McCarty , A. J. Reininger , M. Sugimoto , M. R. King , J. Thromb. Haemostasis 2014, 12, 418. 24330648 10.1111/jth.12482

[29] T. Wen‐Hsiang , Sci. Direct. 1985, 29, 377.

[30] M. Ranucci , A. Ballotta , U. Di Dedda , E. Bayshnikova , M. Dei Poli , M. Resta , M. Falco , G. Albano , L. Menicanti , J. Thromb. Haemostasis 2020, 18, 1747. 32302448 10.1111/jth.14854 PMC9906332

[31] G. Zhou , J. Xia , Curr. Protoc. Bioinf. 2019, 65, e69. 10.1002/cpbi.69 30556956

[32] J. Wu , S. Song , H. C. Cao , L. J. Li , World J. Gastroenterol. 2020, 26, 2286. 32476793 10.3748/wjg.v26.i19.2286 PMC7243650

[33] B. Bikdeli , M. V. Madhavan , D. Jimenez , T. Chuich , I. Dreyfus , E. Driggin , C. Nigoghossian , W. Ageno , M. Madjid , Y. Guo , L. V. Tang , Y. Hu , J. Giri , M. Cushman , I. Quere , E. P. Dimakakos , C. M. Gibson , G. Lippi , E. J. Favaloro , J. Fareed , J. A. Caprini , A. J. Tafur , J. R. Burton , D. P. Francese , E. Y. Wang , A. Falanga , C. McLintock , B. J. Hunt , A. C. Spyropoulos , G. D. Barnes , J. W. Eikelboom , I. Weinberg , S. Schulman , M. Carrier , G. Piazza , J. A. Beckman , P. G. Steg , G. W. Stone , S. Rosenkranz , S. Z. Goldhaber , S. A. Parikh , M. Monreal , H. M. Krumholz , S. V. Konstantinides , J. I. Weitz , G. Y. H. Lip , J. Am. Coll. Cardiol. 2020, 75, 2950. 32311448 10.1016/j.jacc.2020.04.031 PMC7164881

[34] N. H. Schultz , I. H. Sorvoll , A. E. Michelsen , L. A. Munthe , F. Lund‐Johansen , M. T. Ahlen , M. Wiedmann , A. H. Aamodt , T. H. Skattor , G. E. Tjonnfjord , P. A. Holme , N. Engl. J. Med. 2021, 384, 2124. 33835768 10.1056/NEJMoa2104882 PMC8112568

[35] Y. Dong , T. Dai , Y. Wei , L. Zhang , M. Zheng , F. Zhou , Signal Transduction Targeted Ther. 2020, 5, 237. 10.1038/s41392-020-00352-y PMC7551521 33051445

[36] M. Li , K. R. Stenmark , R. Shandas , W. Tan , J. Vasc. Res. 2009, 46, 561. 19571576 10.1159/000226224 PMC3073484

[37] B. J. Ballermann , A. Dardik , E. Eng , A. Liu , Kidney Int. Suppl. 1998, 67, S100. 9736263 10.1046/j.1523-1755.1998.06720.x

[38] J. Song , B. Hu , H. Qu , L. Wang , X. Huang , M. Li , M. Zhang , Biochem. Biophys. Res. Commun. 2020, 525, 812. 32169277 10.1016/j.bbrc.2020.02.151

[39] O. D. Rotstein , Eur. J. Clin. Microbiol. Infect. Dis. 1992, 11, 1064. 1295760 10.1007/BF01967800

[40] I. K. Mullarky , F. M. Szaba , K. N. Berggren , M. A. Parent , L. W. Kummer , W. Chen , L. L. Johnson , S. T. Smiley , Infect. Immun. 2005, 73, 3888. 15972474 10.1128/IAI.73.7.3888-3895.2005 PMC1168549

[41] J. D. McFadyen , H. Stevens , K. Peter , Circ. Res. 2020, 127, 571. 32586214 10.1161/CIRCRESAHA.120.317447 PMC7386875

[42] A. Rubini , Inflammation Allergy: Drug Targets 2013, 12, 315. 23859697 10.2174/1871528111312050003

[43] C. Sardu , J. Gambardella , M. B. Morelli , X. Wang , R. Marfella , G. Santulli , J. Clin. Med. 2020, 9, 9. 10.3390/jcm9051417 PMC7290769 32403217

[44] Y. Liu , Y. Yang , C. Zhang , F. Huang , F. Wang , J. Yuan , Z. Wang , J. Li , J. Li , C. Feng , Z. Zhang , L. Wang , L. Peng , L. Chen , Y. Qin , D. Zhao , S. Tan , L. Yin , J. Xu , C. Zhou , C. Jiang , L. Liu , Sci. China: Life Sci. 2020, 63, 364. 32048163 10.1007/s11427-020-1643-8 PMC7088566

[45] M. E. Mehrabadi , R. Hemmati , A. Tashakor , A. Homaei , M. Yousefzadeh , K. Hemati , S. Hosseinkhani , Biomed. Pharmacother. 2021, 137, 111363. 33582450 10.1016/j.biopha.2021.111363 PMC7862910

[46] E. Lindmark , E. Diderholm , L. Wallentin , A. Siegbahn , JAMA, J. Am. Med. Assoc. 2001, 286, 2107. 10.1001/jama.286.17.2107 11694151

[47] J. Aulin , A. Siegbahn , Z. Hijazi , M. D. Ezekowitz , U. Andersson , S. J. Connolly , K. Huber , P. A. Reilly , L. Wallentin , J. Oldgren , Am. Heart J. 2015, 170, 1151. 26678637 10.1016/j.ahj.2015.09.018

[48] X. Chen , B. Zhao , Y. Qu , Y. Chen , J. Xiong , Y. Feng , D. Men , Q. Huang , Y. Liu , B. Yang , J. Ding , F. Li , Clin. Infect. Dis. 2020, 71, 1937. 32301997 10.1093/cid/ciaa449 PMC7184354

[49] L. Y. C. Chen , R. L. Hoiland , S. Stukas , C. L. Wellington , M. S. Sekhon , Eur. Respir. J. 2020, 56. 10.1183/13993003.03006-2020 PMC7474149 32883678

[50] I. O. Rosas , N. Brau , M. Waters , R. C. Go , B. D. Hunter , S. Bhagani , D. Skiest , M. S. Aziz , N. Cooper , I. S. Douglas , S. Savic , T. Youngstein , L. Del Sorbo , A. Cubillo Gracian , D. J. De La Zerda , A. Ustianowski , M. Bao , S. Dimonaco , E. Graham , B. Matharu , H. Spotswood , L. Tsai , A. Malhotra , N. Engl. J. Med. 2021, 384, 1503. 33631066 10.1056/NEJMoa2028700 PMC7953459

[51] R.‐C. Investigators , A. C. Gordon , P. R. Mouncey , F. Al‐Beidh , K. M. Rowan , A. D. Nichol , Y. M. Arabi , D. Annane , A. Beane , W. van Bentum‐Puijk , L. R. Berry , Z. Bhimani , M. J. M. Bonten , C. A. Bradbury , F. M. Brunkhorst , A. Buzgau , A. C. Cheng , M. A. Detry , E. J. Duffy , L. J. Estcourt , M. Fitzgerald , H. Goossens , R. Haniffa , A. M. Higgins , T. E. Hills , C. M. Horvat , F. Lamontagne , P. R. Lawler , H. L. Leavis , K. M. Linstrum , E. Litton , E. Lorenzi , J. C. Marshall , F. B. Mayr , D. F. McAuley , A. McGlothlin , S. P. McGuinness , B. J. McVerry , S. K. Montgomery , S. C. Morpeth , S. Murthy , K. Orr , R. L. Parke , J. C. Parker , A. E. Patanwala , V. Pettila , E. Rademaker , M. S. Santos , C. T. Saunders , C. W. Seymour , M. Shankar‐Hari , W. I. Sligl , A. F. Turgeon , A. M. Turner , F. L. van de Veerdonk , R. Zarychanski , C. Green , R. J. Lewis , D. C. Angus , C. J. McArthur , S. Berry , S. A. Webb , L. P. G. Derde , N. Engl. J. Med. 2021, 384, 1491. 33631065 10.1056/NEJMoa2100433 PMC7953461

[52] Anti‐il6 treatment of serious covid‐19 disease with threatening respiratory failure (tocivid). 2021. https://www.clinicaltrials.gov/ct2/show/NCT04322773

