# Supplementary material for: Rapid Detection and Inhibition of SARS‐CoV‐2‐Spike Mutation‐Mediated Microthrombosis
Source: Adv Sci (Weinh). 2021 Oct 23;8(23):2103266. doi: 10.1002/advs.202103266 (PMC8646611; doi:10.1002/advs.202103266)
Supplement: Supplementary file 1 — Supporting Information [file ADVS-8-2103266-s001.pdf]

Rapid Detection and Inhibition of SARS-CoV-2-Spike Mutation-Mediated Microthrombosis

S1

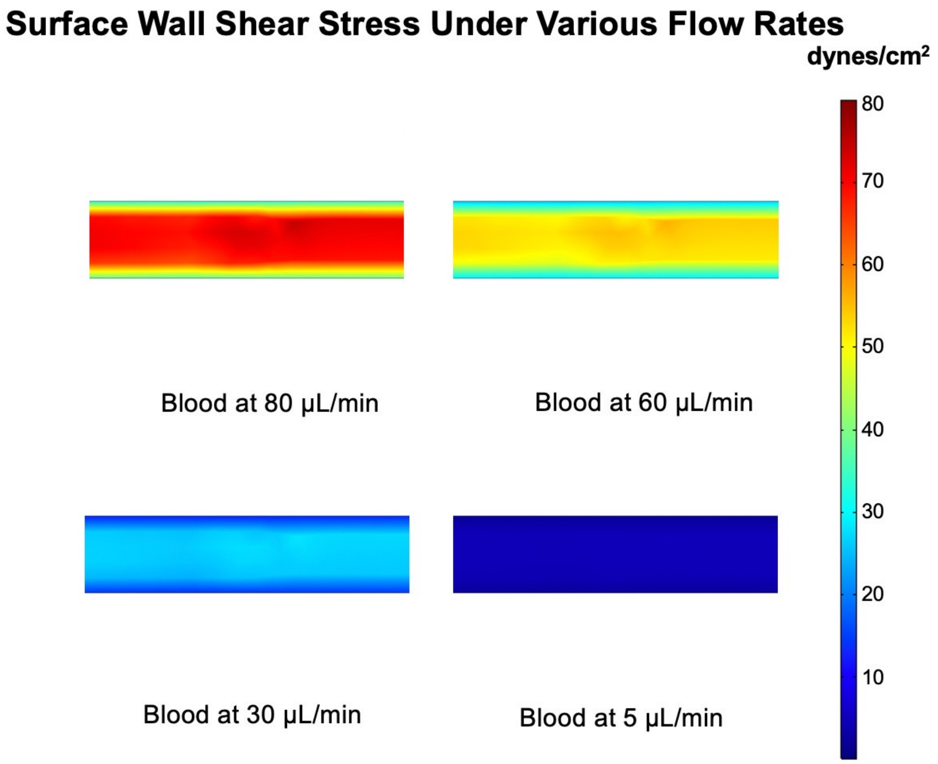

Figure S1. Computational fluid dynamic simulations of wall shear stress in the microfluidic channel at varying flow rates was performed in COMSOL to select a flow rate with a wall shear stress of 25 dyne/cm^2. The simulation with a flow rate of 30 μL/min provided the desired wall shear stress and was selected for use in the benchtop experiments.

S2

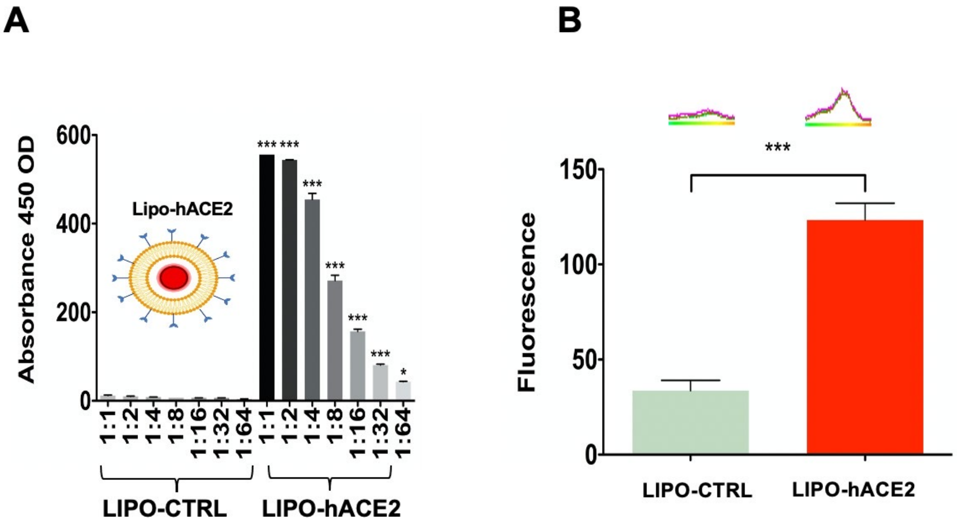

Figure S2. (**A**) Serial dilutions of the Liposome-hACE2 complex were evaluated with indirect ELISA test (absorbance 450 OD) (*$p < 0.05$ and *** $p < 0.001$ vs. corresponding Lipo-CTRL, n = 8). (**B)** Dilution factor at 1:32 was used to confirm the binding between the liposomes encapsulated with rhodamine and the biotinylated-hACE2 protein (Fluorescence Ex 546 nm and Em 568 nm) (***$p < 0.001$ vs. Lipo-CTRL, n=8).

S3

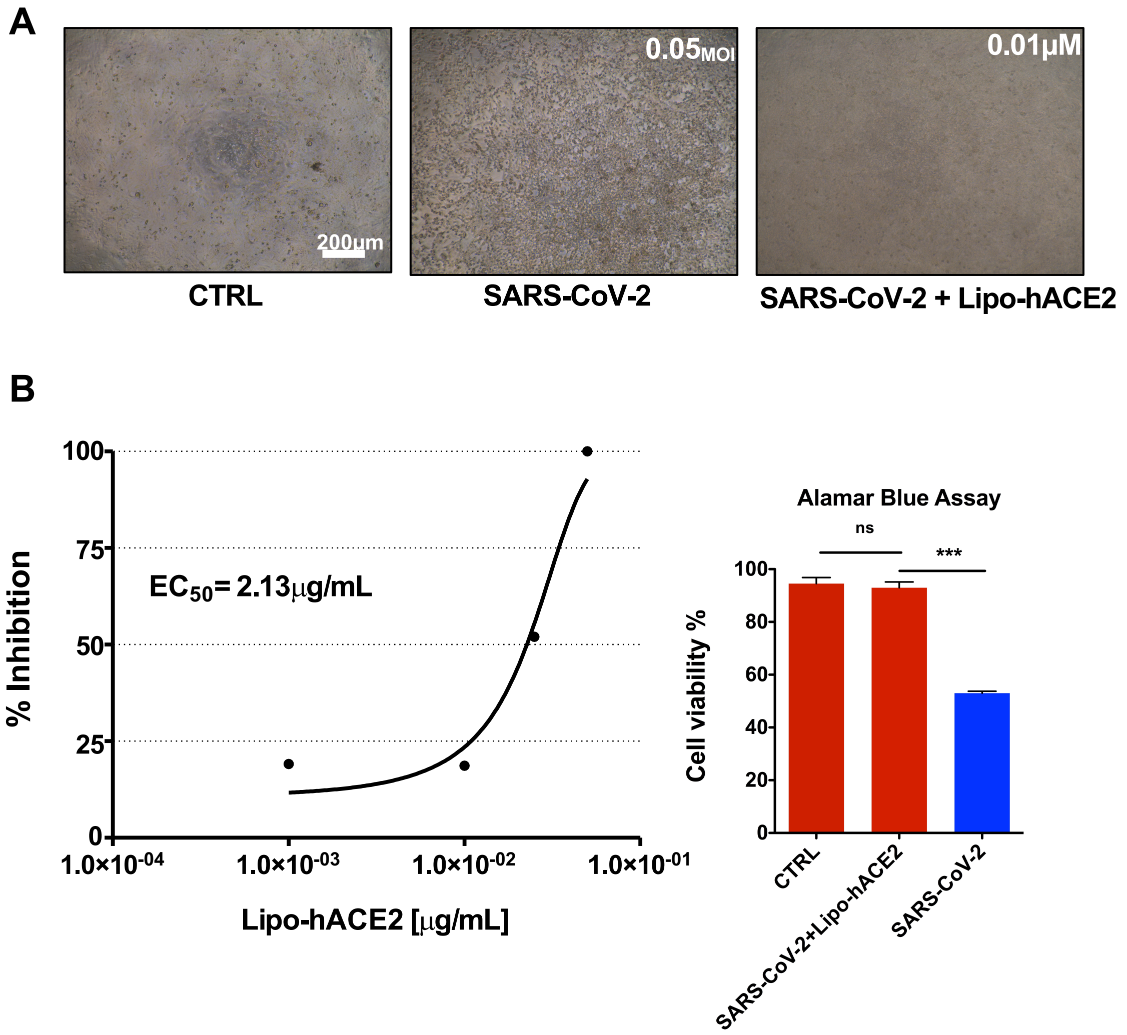

Figure S3. (A) Lipo-hACE2 inhibits SARS-CoV-2 binding to the ACE2 receptors, hence, mitigating its cytopathic effects. **(B)** Viral RNA extraction and quantitative real-time RT-PCR was performed to evaluate the SARS-CoV-2 replication by Lipo-hACE2-mediated inhibition. Dose-dependent curve (0.085, 0.085, 2.13, 4.27µg/mL respectively) showed EC_{50}=2.13 µg/mL (n=3). Data are presented as median (n=3). (**C)** Viability of SARS-CoV-2-infected cells (0.05 $_{MOI}$) at 48 hours was measured using Alamar Blue viability assay. Viability was compared to the non-infected control cells (600 nm Absorbance). 0.85 µg/mL of Lipo-hACE2 efficiently inhibited SARS-CoV-2-infected cells (****p*<0.001 CTRL or Lipo-hACE2 + SARS-CoV-2 vs. SARS-CoV-2 only, n=5).

S4

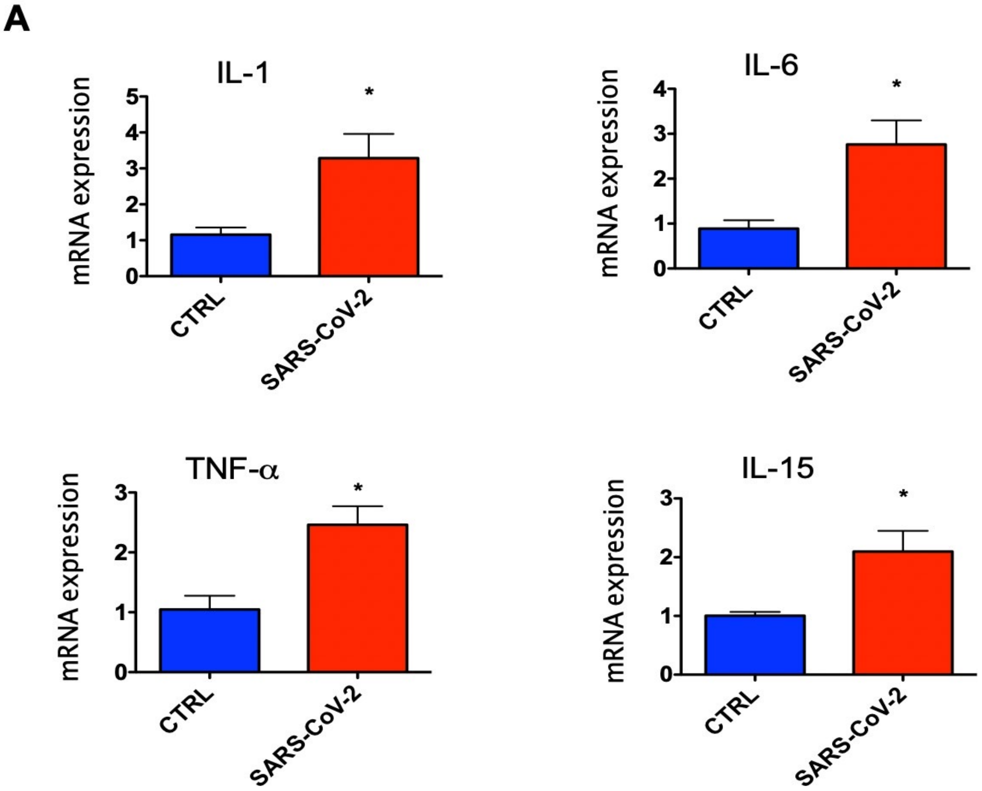

Figure S4. SARS-CoV-2-induced cytokine mRNA expression. TNF-α, IL-1, IL-6 and IL-15 were significantly upregulated (*$p < 0.05$, *t*-test CTRL vs. SARS-CoV-2 infected cells, n=3).

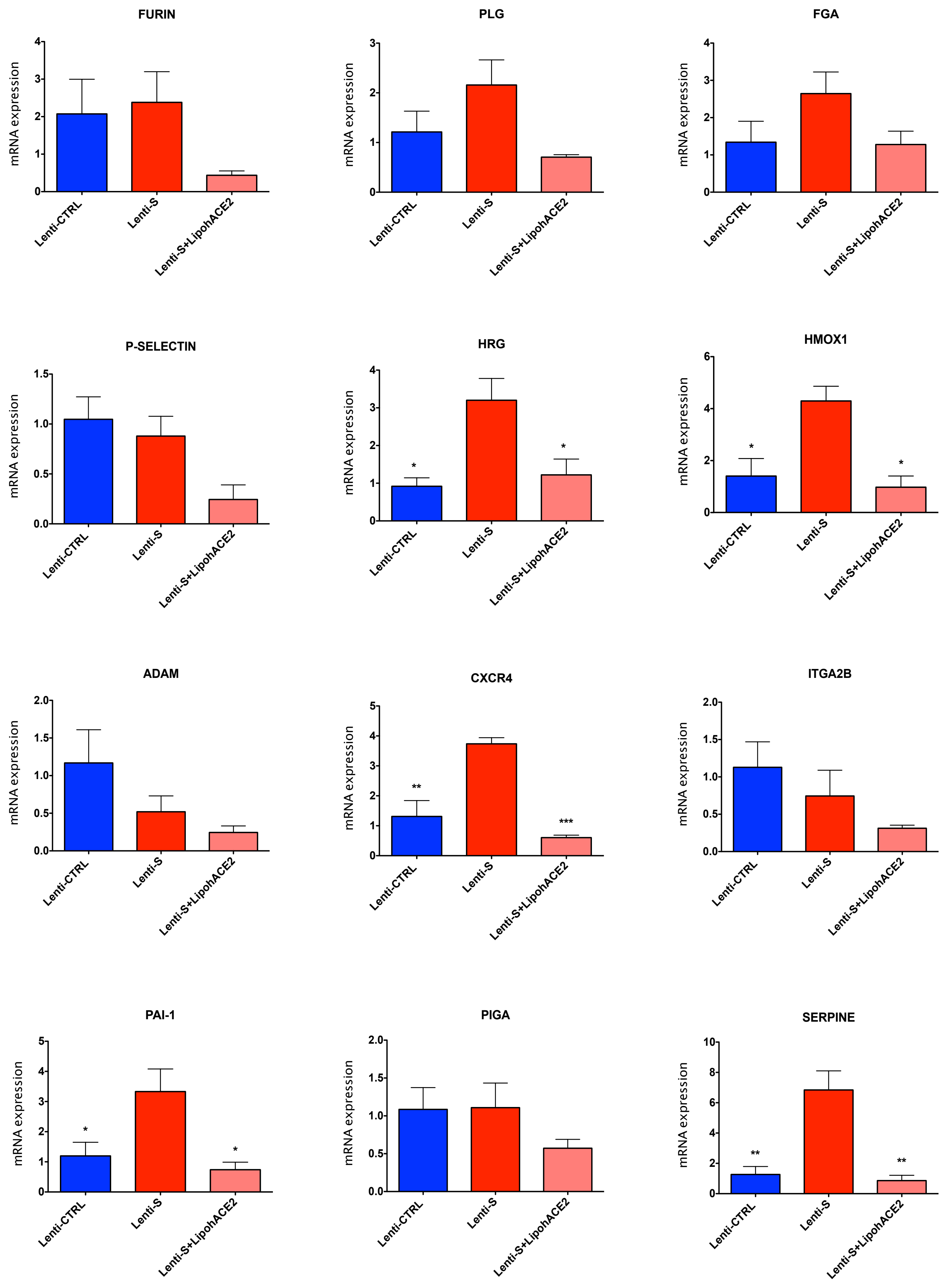
FURIN
PLG
FGA
P-SELECTIN
HRG
HMOX1
ADAM
CXCR4
ITGA2B
PAI-1
PIGA
SERPINE
mRNA expression
Lenti-CTRL
Lenti-S
Lenti-S+LipohACE2
*
**
***

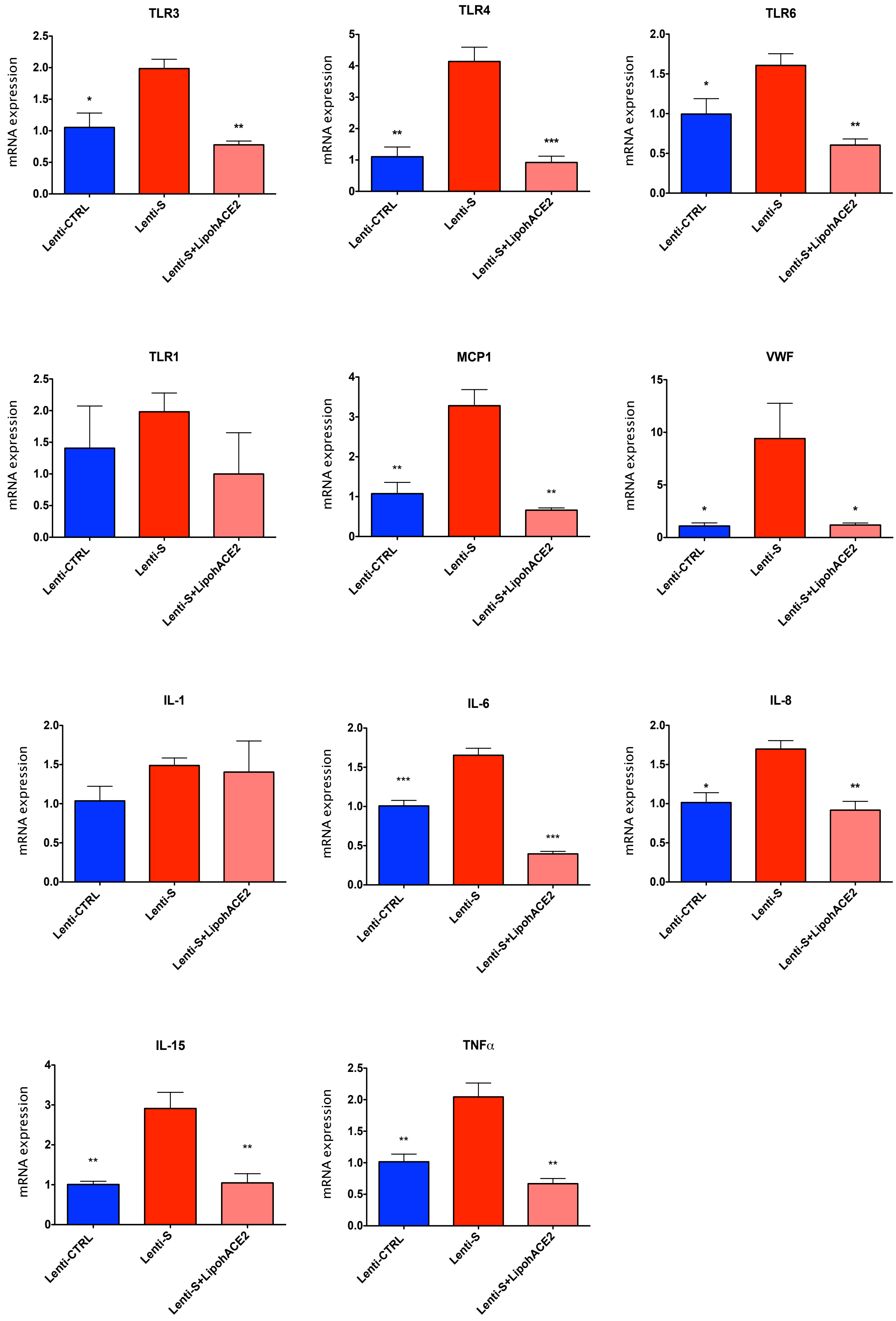
TLR3
TLR4
TLR6
TLR1
MCP1
VWF
IL-1
IL-6
IL-8
IL-15
TNFα
mRNA expression
0.0
0.5
1.0
1.5
2.0
2.5
0
1
2
3
4
5
10
15
*
**
***
Lenti-CTRL
Lenti-S
Lenti-S+LipohACE2

Figure S5. Lenti-S mutation D614G upregulated mRNA expression of cytokines, chemokines, and thrombotic genes in HAECS. In the presence Lipo-hACE2, mRNA expression these same cytokines, chemokines, and thrombotic genes were downregulated when compared to the control (* $p < 0.05$, ** $p < 0.01$, *** $p < 0.001$, Lenti-CTRL and Lenti-S mutation + Lipo-hACE2 vs. Lenti-S mutation, n=3).

Table S1. Gene ontology (GO) analysis was used to reveal the biological significance of the differentially expressed genes. We demonstrated a gene set enrichment analysis, revealing 3 significantly enriched GO terms (*p*-value < 0.01): biological processes, molecular function, and cellular components.

Pathway	Total	Expected	Hits	P.Value	FDR
Toll-Like Receptors Cascades	123	5.66	51	7.16E-37	1E-33
Activated TLR4 signalling	100	4.6	46	8.8E-36	6.17E-33
Toll Like Receptor 4 (TLR4) Cascade	103	4.74	46	4.72E-35	2.21E-32
TRIF-mediated TLR3/TLR4 signaling	87	4	38	1.4E-28	4.92E-26
MyD88-independent cascade	88	4.05	38	2.37E-28	5.54E-26
Toll Like Receptor 3 (TLR3) Cascade	88	4.05	38	2.37E-28	5.54E-26
Immune System	1140	52.3	130	8.25E-27	1.65E-24
Signaling by Interleukins	116	5.33	41	2.04E-26	3.57E-24
MyD88:Mal cascade initiated on plasma membrane	81	3.72	35	3.53E-26	4.12E-24
Toll Like Receptor TLR6:TLR2 Cascade	81	3.72	35	3.53E-26	4.12E-24
Innate Immune System	521	24	82	6.16E-25	6.65E-23
Toll Like Receptor 10 (TLR10) Cascade	74	3.4	28	3.1E-19	2.56E-17
Toll Like Receptor 5 (TLR5) Cascade	74	3.4	28	3.1E-19	2.56E-17
MyD88 cascade initiated on plasma membrane	74	3.4	28	3.1E-19	2.56E-17
NOD1/2 Signaling Pathway	38	1.75	21	5.93E-19	4.62E-17
Platelet degranulation	89	4.09	30	7.3E-19	5.38E-17
Response to elevated platelet cytosolic Ca2+	94	4.32	30	4.27E-18	2.99E-16
Hemostasis	511	23.5	70	1.16E-17	7.76E-16
Platelet activation, signaling and aggregation	220	10.1	44	2.45E-17	1.56E-15
Nucleotide-binding domain, leucine rich repeat containing receptor (NLR) signaling pathways	55	2.53	23	3.79E-17	2.31E-15
Cytokine Signaling in Immune system	286	13.1	50	4.95E-17	2.89E-15
TRAF6 mediated induction of NFkB and MAP kinases upon TLR7/8 or 9 activation	76	3.49	26	1.14E-16	6.4E-15
Toll Like Receptor 7/8 (TLR7/8) Cascade	77	3.54	26	1.65E-16	8.58E-15
MyD88 dependent cascade initiated on endosome	77	3.54	26	1.65E-16	8.58E-15
Toll Like Receptor 9 (TLR9) Cascade	79	3.63	26	3.4E-16	1.7E-14
IKK complex recruitment mediated by RIP1	25	1.15	16	3.82E-16	1.85E-14
TAK1 activates NFkB by phosphorylation and activation of IKKs complex	22	1.01	15	7.92E-16	3.7E-14
TRAF6 Mediated Induction of proinflammatory cytokines	62	2.85	23	9.03E-16	4.09E-14
TRAF6 mediated induction of TAK1 complex	16	0.736	13	1.59E-15	6.95E-14
Activation of IRF3/IRF7 mediated by TBK1/IKK epsilon	16	0.736	11	5.8E-12	2.46E-10
MAP kinase activation in TLR cascade	55	2.53	18	1.58E-11	6.52E-10
TRIF-mediated programmed cell death	11	0.506	9	4.16E-11	1.66E-09
activated TAK1 mediates p38 MAPK activation	23	1.06	12	6.2E-11	2.41E-09
GRB2:SOS provides linkage to MAPK signaling for Intergrins	15	0.69	10	8.95E-11	3.39E-09
DAI mediated induction of type I IFNs	13	0.598	9	4.98E-10	1.84E-08
Chemokine receptors bind chemokines	59	2.71	17	5.67E-10	2.04E-08
RIG-I/MDA5 mediated induction of IFN-alpha/beta pathways	67	3.08	18	6.21E-10	2.18E-08
Formation of Fibrin Clot (Clotting Cascade)	29	1.33	12	1.85E-09	6.34E-08
Cytosolic sensors of pathogen-associated DNA	19	0.874	10	2.34E-09	7.79E-08
p130Cas linkage to MAPK signaling for integrins	15	0.69	9	3.21E-09	1.05E-07
JNK (c-Jun kinases) phosphorylation and activation mediated by activated human TAK1	20	0.92	10	4.48E-09	1.43E-07
Integrin cell surface interactions	85	3.91	19	6E-09	1.87E-07
Integrin alphaIIb beta3 signaling	27	1.24	11	1.1E-08	3.34E-07
Platelet Aggregation (Plug Formation)	36	1.66	12	3.34E-08	9.96E-07
Dissolution of Fibrin Clot	10	0.46	7	4.33E-08	1.21E-06
IRAK2 mediated activation of TAK1 complex upon TLR7/8 or 9 stimulation	10	0.46	7	4.33E-08	1.21E-06
IRAK2 mediated activation of TAK1 complex	10	0.46	7	4.33E-08	1.21E-06
TNF signaling	7	0.322	6	6.07E-08	1.67E-06
Cell surface interactions at the vascular wall	99	4.55	19	8.39E-08	2.26E-06
RIP-mediated NFkB activation via DAI	11	0.506	7	1.14E-07	3.02E-06
Downstream TCR signaling	48	2.21	13	1.44E-07	3.73E-06
p75NTR signals via NF-kB	16	0.736	8	1.7E-07	4.26E-06
TRAF6 mediated NF-kB activation	16	0.736	8	1.7E-07	4.26E-06
Death Receptor Signalling	12	0.552	7	2.64E-07	6.37E-06
Extrinsic Pathway for Apoptosis	12	0.552	7	2.64E-07	6.37E-06
TCR signaling	65	2.99	14	1.02E-06	2.43E-05
Interleukin-6 signaling	14	0.644	7	1.05E-06	2.46E-05
Signaling by the B Cell Receptor (BCR)	199	9.15	26	1.14E-06	2.62E-05
Signal regulatory protein (SIRP) family interactions	20	0.92	8	1.42E-06	3.2E-05

Table S1. Continued

EGFR downregulation	27	1.24	9	1.84E-06	4.09E-05
Interleukin-3, 5 and GM-CSF signaling	51	2.34	12	2.27E-06	4.98E-05
Adaptive Immune System	654	30.1	56	2.39E-06	5.14E-05
IRAK1 recruits IKK complex upon TLR7/8 or 9 stimulation	16	0.736	7	3.24E-06	6.79E-05
IRAK1 recruits IKK complex	16	0.736	7	3.24E-06	6.79E-05
Common Pathway	11	0.506	6	3.42E-06	7.05E-05
Intrinsic Pathway	17	0.782	7	5.3E-06	0.000108
Activation of NF-kappaB in B Cells	66	3.03	13	7.19E-06	0.000144
Signal Transduction	1690	77.6	111	9.75E-06	0.000193
NF-kB is activated and signals survival	13	0.598	6	1.17E-05	0.000226
p75NTR recruits signalling complexes	13	0.598	6	1.17E-05	0.000226
Interleukin-2 signaling	42	1.93	10	1.44E-05	0.000274
Signal transduction by L1	35	1.61	9	2E-05	0.000374
Peptide ligand-binding receptors	192	8.83	23	2.07E-05	0.000382
CaMK IV-mediated phosphorylation of CREB	5	0.23	4	2.11E-05	0.000382
Dimerization of procaspase-8	9	0.414	5	2.15E-05	0.000382
Caspase-8 activation	9	0.414	5	2.15E-05	0.000382
Signalling by NGF	290	13.3	30	2.19E-05	0.000383
Interleukin receptor SHC signaling	28	1.29	8	2.53E-05	0.000438
Developmental Biology	417	19.2	38	3.2E-05	0.000546
Downstream Signaling Events Of B Cell Receptor (BCR)	173	7.95	21	3.92E-05	0.000662
Interferon alpha/beta signaling	68	3.13	12	5.16E-05	0.00086
Activation of Matrix Metalloproteinases	31	1.43	8	5.69E-05	0.000938
CREB phosphorylation through the activation of CaMKK	6	0.276	4	6.11E-05	0.000996
TRAF6 mediated IRF7 activation in TLR7/8 or 9 signaling	17	0.782	6	7.24E-05	0.00117
Apoptosis	158	7.26	19	0.000104	0.00166
Interleukin-7 signaling	12	0.552	5	0.000121	0.00187
NF-kB activation through FADD/RIP-1 pathway mediated by caspase-8 and -10	12	0.552	5	0.000121	0.00187
Interferon gamma signaling	74	3.4	12	0.000121	0.00187
Signaling by PDGF	189	8.69	21	0.000143	0.00218
PECAM1 interactions	13	0.598	5	0.000189	0.00284
Signaling by EGFR	179	8.23	20	0.000191	0.00285
SMAD2/SMAD3:SMAD4 heterotrimer regulates transcription	28	1.29	7	0.000208	0.00307
Signaling by EGFR in Cancer	181	8.32	20	0.000222	0.00325
Regulation of Insulin-like Growth Factor (IGF) Transport and Uptake by Insulin-like Growth Fac	21	0.966	6	0.000272	0.00393
TRAF3-dependent IRF activation pathway	14	0.644	5	0.000283	0.00404
Signaling by TGF-beta Receptor Complex	70	3.22	11	0.000309	0.00437
MAPK targets/ Nuclear events mediated by MAP kinases	30	1.38	7	0.000331	0.00464
Activation of CaMK IV	4	0.184	3	0.000372	0.00516
NOTCH1 Intracellular Domain Regulates Transcription	50	2.3	9	0.000386	0.00531
eNOS activation	9	0.414	4	0.00046	0.00626
Activation of the pre-replicative complex	32	1.47	7	0.000505	0.00675
Antigen Activates B Cell Receptor Leading to Generation of Second Messengers	32	1.47	7	0.000505	0.00675
Assembly of the pre-replicative complex	63	2.9	10	0.000532	0.00703
Signaling by ERBB4	152	6.99	17	0.000569	0.0074
L1CAM interactions	112	5.15	14	0.00057	0.0074
GPVI-mediated activation cascade	33	1.52	7	0.000616	0.00793
Regulation of mRNA Stability by Proteins that Bind AU-rich Elements	88	4.05	12	0.000637	0.00812
Transcriptional activity of SMAD2/SMAD3:SMAD4 heterotrimer	43	1.98	8	0.000646	0.00816
Activation of the AP-1 family of transcription factors	10	0.46	4	0.000738	0.00916
GP1b-IX-V activation signalling	10	0.46	4	0.000738	0.00916
Signaling by SCF-KIT	142	6.53	16	0.000758	0.00932
Regulation of IFNG signaling	17	0.782	5	0.000779	0.0095
Extracellular matrix organization	157	7.22	17	0.000826	0.00998
Activation of Chaperone Genes by ATF6-alpha	5	0.23	3	0.000898	0.0108
DNA Replication Pre-Initiation	80	3.68	11	0.000993	0.0117
M/G1 Transition	80	3.68	11	0.000993	0.0117
Switching of origins to a post-replicative state	69	3.17	10	0.00111	0.0127
